# Anesthetic Management of Pregnant Patient With Undiagnosed Neurofibromatosis for Emergency Cesarean Section

**DOI:** 10.7759/cureus.34786

**Published:** 2023-02-08

**Authors:** Swathi Mallikarjuna, Deepika Karjigi, Sakshi Kadian, Ajit Kumar

**Affiliations:** 1 Anesthesiology, All India Institute of Medical Sciences, Rishikesh, IND

**Keywords:** multisystem, airway, anesthesia, neurofibromatosis, pregnant

## Abstract

Neurofibromatosis type I is a genetic autosomal dominant disorder with multisystem involvement and is particularly challenging for the anesthesiologist in emergency surgery. The presence of neurofibromas can cause airway difficulty, make delivery of gases difficult, and blood pressure variations during general anesthesia. Regional anesthesia becomes challenging due to the presence of spinal and intracranial tumors, and in undiagnosed situations, it becomes tricky. This is a case report of anesthesia management in a pregnant patient with undiagnosed neurofibromatosis for an emergency cesarean section.

## Introduction

Neurofibromatosis type I is a multisystem autosomal dominant neurocutaneous disorder caused by the mutation of a gene located on chromosome 17q11.2 encoding the neurofibromin gene [1]. This is particularly challenging to the anesthesiologist due to multisystem involvement [2], and complications of the disorder while evaluating the patient for surgical procedures. In emergency surgery, the thorough evaluation of the patient is not feasible and makes the management of the anesthesia plan difficult. Here we present a case report of anesthesia management of a 26-year-old pregnant female with undiagnosed neurofibromatosis for emergency cesarean section.

## Case presentation

**Figure 1:**
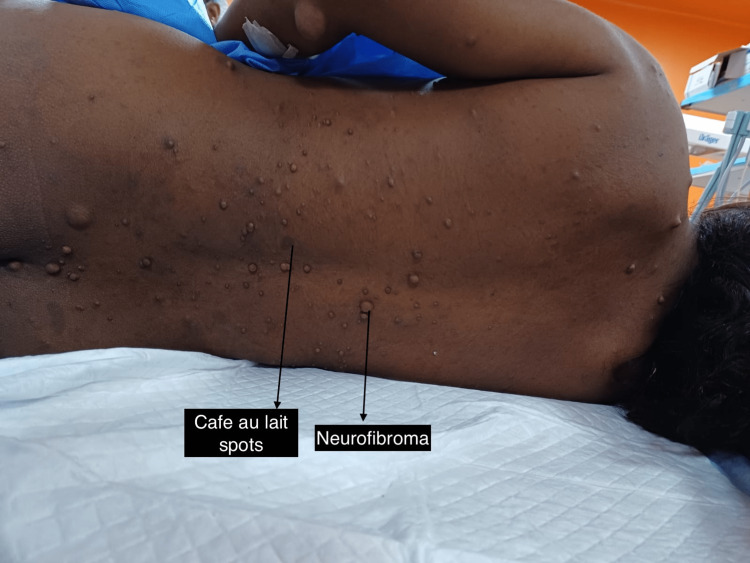
Multiple cafe au lait spots and neurofibroma on the back

**Figure 2:**
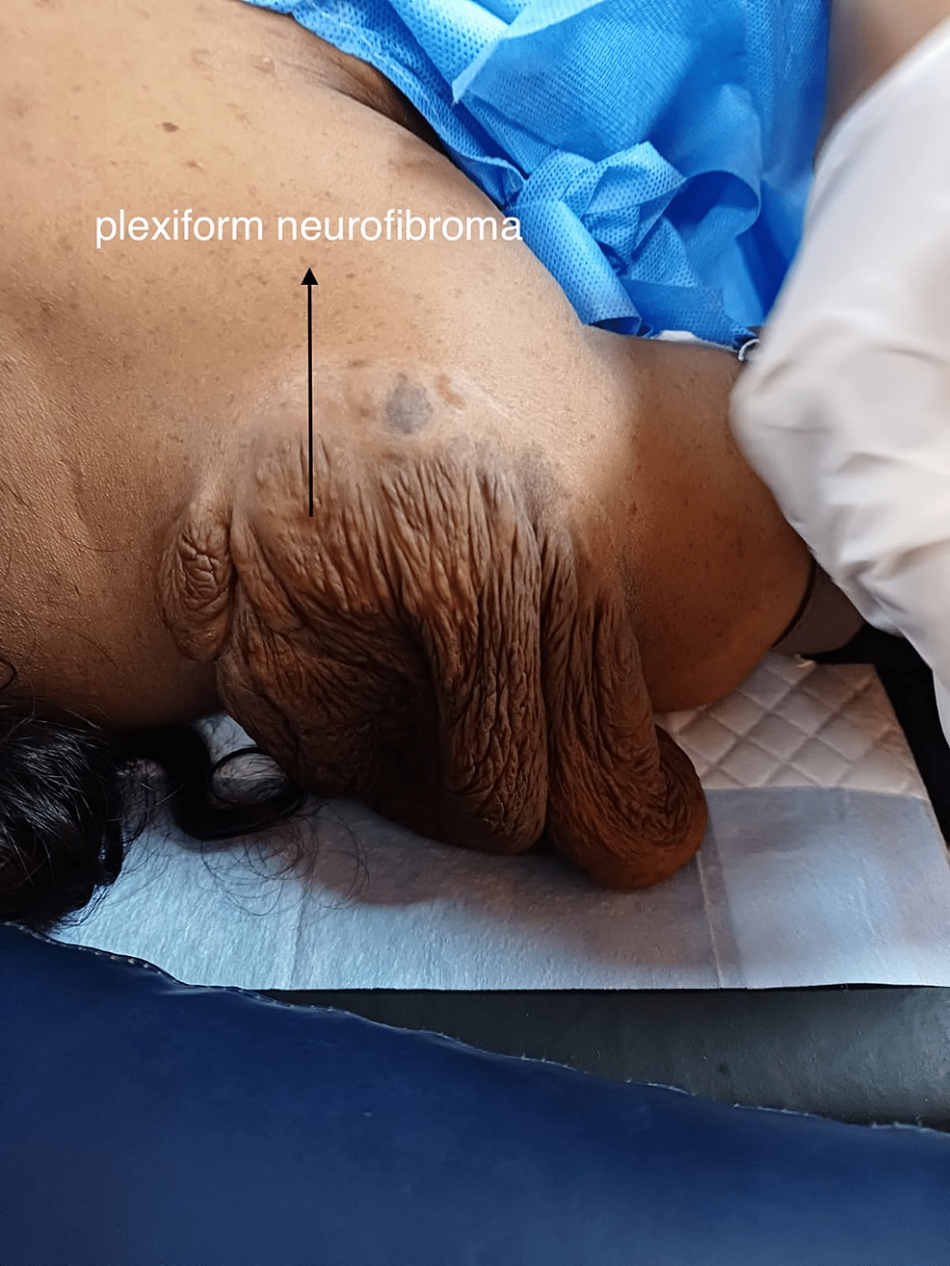
Plexiform Neurofibroma over the right shoulder

A 26-year-old female of G5P4L4 with previous four vaginal deliveries was referred to our hospital in view of anemia and meconium-stained liquor for emergency cesarean section. On preoperative evaluation, there was the presence of multiple swellings and café au lait spots on the body (Figure 1) since birth, a large plexiform neurofibroma in the right shoulder area (Figure 2), and a neurofibroma in the oral cavity. She also had axillary freckling. On eye examination, she was observed to have an irisch nodule. She was not evaluated and diagnosed for the same before as her previous four vaginal deliveries were at home. Based on the history and clinical features, a clinical diagnosis of neurofibromatosis type I was made and taken up for emergency cesarean section in view of meconium-stained liquor. Since the patient was not thoroughly evaluated and no investigation has been done for neurofibromatosis as it was not diagnosed and documented before, it was decided for the emergency cesarean section to be done under general anesthesia as spinal anesthesia will be risky due to the possibility of unknown spinal and intracranial tumors. After the attachment of standard anesthesia monitors, rapid sequence induction was done with an injection of propofol 100mg IV and succinylcholine 100mg IV. There was blood pressure variation intraoperatively managed with fluids and esmolol. After the delivery of the baby, opioids were given. The rest of the perioperative events was uneventful. The patient was extubated on the table and shifted to the ICU for further monitoring.

## Discussion

**Figure 3:**
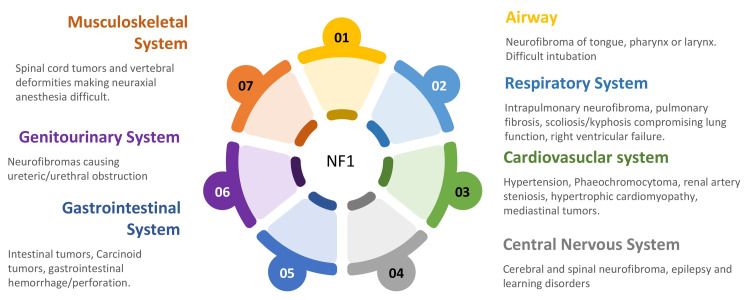
Multisystemic involvement in neurofibromatosis type 1 NF1 : Neurofibromatosis type 1 Figure created by the authors

Neurofibromatosis was first reviewed and published in the year 1849 by Robert W Smith [3]. In 1882, Freidrich von Recklinghausen first recognized the origin of the tumor from nervous tissue [4], and hence also known as von Recklinghausen disease. It is classified into two types. Type 1 is more common and involves multisystem. Diagnostic criteria for type 1 were developed by the National Institute of Health Consensus Conference in 1987 and are based on clinical findings [5]. The multisystemic involvement complicates anesthesia management (Figure 3) and is more challenging in patients not evaluated as in our case. Though there are reports of spinal anesthesia successfully given in a pregnant patient with neurofibromatosis, it is only done after thorough evaluation [6,7]. Since unknown intracranial tumors and spinal neuromas which can be seen in 40% of cases [8]^ ^complicate the neurological picture under regional neuraxial anesthesia, general anesthesia is a safer option. As the patient was undiagnosed for neurofibromatosis, we proceeded with general anesthesia for an emergency cesarean section.

The anesthetic implications in NF1 are difficult airway, oral neurofibromas as in our case, supraglottic neurofibromas causing airway obstruction and delivery of respiratory gases difficult, occult pheochromocytoma and intraoperative hypertension as seen in our case. Other considerations are respiratory compromise due to kyphoscoliosis, intrapulmonary fibrosis, and decreased cardiopulmonary reserve [9].

Intraoperative hypertension seen in our case may be due to occult pheochromocytoma, which was managed with beta-blockers and the patient was stable perioperatively. Some patients may have altered sensitivity to neuromuscular blockers [10], which was not seen in our case. Keeping in view that general anesthesia is safer than neuraxial anesthesia in emergencies where necessary investigations are not done, this case was uneventful concerning the preparation for difficult airway and hemodynamic variation.

## Conclusions

A pregnancy associated with neurofibromatosis must be evaluated thoroughly preoperatively, and physical examination and imaging methods should be done. If these are not possible in emergency situations, then general anesthesia with a titrated dose of anesthetic agents will be a safer option with necessary precautions taken for difficult airway, hemodynamic variations, and multisystemic complications.
